# Using microneedle array electrodes for non-invasive electrophysiological signal acquisition and sensory feedback evoking

**DOI:** 10.3389/fbioe.2023.1238210

**Published:** 2023-08-03

**Authors:** Xi Tang, Yuanzhe Dong, Qingge Li, Zhiyuan Liu, Nan Yan, Yongcheng Li, Bin Liu, Lelun Jiang, Rong Song, Yingying Wang, Guanglin Li, Peng Fang

**Affiliations:** ^1^ CAS Key Laboratory of Human-Machine Intelligence-Synergy Systems, Shenzhen Institute of Advanced Technology & Shenzhen Engineering Laboratory of Neural Rehabilitation Technology, Shenzhen, China; ^2^ Shenzhen College of Advanced Technology, University of Chinese Academy of Sciences, Shenzhen, China; ^3^ Guangdong Provincial Key Laboratory of Sensor Technology and Biomedical Instrument, School of Biomedical Engineering, Sun Yat-sen University, Guangzhou, China

**Keywords:** neural interface, microneedle array electrode, electrophysiological, EMG, transcutaneous electrical nerve stimulation, sensory feedback, human-machine interaction

## Abstract

**Introduction:** Bidirectional transmission of information is needed to realize a closed-loop human-machine interaction (HMI), where electrophysiological signals are recorded for man-machine control and electrical stimulations are used for machine-man feedback. As a neural interface (NI) connecting man and machine, electrodes play an important role in HMI and their characteristics are critical for information transmission.

**Methods:** In this work, we fabricated a kind of microneedle array electrodes (MAEs) by using a magnetization-induced self-assembly method, where microneedles with a length of 500–600 μm and a tip diameter of ∼20 μm were constructed on flexible substrates. Part of the needle length could penetrate through the subjects’ stratum corneum and reach the epidermis, but not touch the dermis, establishing a safe and direct communication pathway between external electrical circuit and internal peripheral nervous system.

**Results:** The MAEs showed significantly lower and more stable electrode-skin interface impedance than the metal-based flat array electrodes (FAEs) in various testing scenarios, demonstrating their promising impedance characteristics. With the stable microneedle structure, MAEs exhibited an average SNR of EMG that is more than 30% higher than FAEs, and a motion-intention classification accuracy that is 10% higher than FAEs. The successful sensation evoking demonstrated the feasibility of the MAE-based electrical stimulation for sensory feedback, where a variety of natural and intuitive feelings were generated in the subjects and thereafter objectively verified through EEG analysis.

**Discussion:** This work confirms the application potential of MAEs working as an effective NI, in both electrophysiological recording and electrical stimulation, which may provide a technique support for the development of HMI.

## 1 Introduction

In a closed-loop human-machine interaction (HMI) system, bidirectional information transmission is always needed to ensure the descending control signals from human brain to machine and the ascending feedback signals from machine to human brain (Xu et al., 2021). For example, in a motorized prosthetic hand, surface electromyography (EMG) signals that contain human motor intentions are recorded from an amputee’s residual limb and then decoded as the control commands (Cohen et al., 2010); tactile signals acquired through the artificial skin of prosthetic hand are encoded and thereafter sent to the user’s brain as sensory feedback by stimulating the nervous systems (Song et al., 2021). Thus, high-quality signal recording and precise stimulation both are very crucial to determine the performance of an HMI system. Electrodes or electrode arrays, as a neural interface (NI) connecting human and machine, have been widely used for both electrophysiological signal recording and electrical stimulation, and their fabrication techniques, modeling and simulation, characteristic assessment, and clinical applications are always a research focus in the areas of HMI (Sundaram et al., 2019).

Many types of electrodes are available for either commercial use or scientific research. Generally, wet electrodes, which use conductive hydrogels for good electrical conduction and adherence to human skin, are mostly used benefiting from their excellent signal acquisition performances (Yuk et al., 2019). In another aspect, the use of hydrogels reversely limits the service life of wet electrodes due to the dehydration of gels with time, which gradually increases the electrode-skin interface impedance (EII) and subsequently lower the signal recording quality (Yao and Zhu, 2016). In addition, variation of EII also hinders stable and precise electrical stimulation if wet electrodes are applied. In actual operation, skin allergies caused by hydrogels often occur in some people, which is another shortcoming of wet electrodes (Yao et al., 2022). As a type of electrode owning the advantage of ready-to-use property with no need of conductive hydrogels, dry electrodes may dominate the future in the discipline of electrodes (Kim et al., 2022). The common metal-based dry electrodes (Yang et al., 2022) are very easy to use but at the expense of high and unstable EII, as well as possible disturbance caused by the relative displacement of electrodes during body movements. The air and water permeable, flexible, and easy-to-wear fabric-based dry electrodes (Acar et al., 2019) enable wearable applications for both signal recording and stimulation with high comfort, but they also meet the difficulties of high EII and motion artifacts in practice (Yang et al., 2014). With the development of flexible electronics technology, the newly proposed flexible and stretchable electrodes (Liu et al., 2022) draw more and more attention due to their compliance to muscle contraction, making them very competitive in dynamic scenes like during body motions, but commercial products of this type of electrodes are still not available until some critical problems are solved, e.g., creep property, low consistency, etc.

Microneedle Array Electrode (MAE) (Ren et al., 2020) is another type of dry electrode with a specifically designed structure. The well-designed microneedle array on the substrate can penetrate through the human stratum corneum and establish a direct connection between the electrodes and the epidermis, without touching the dermis that contains blood capillaries and nerve endings. Thus, electrophysiological signals are recorded by eliminating the influence of stratum corneum that shows high impedance, and possible injury to the dermis or even to the whole body would be avoided, which is the most disadvantage of invasive methods for signal recording. In addition, the microneedles embedded in the skin can fix the whole device firmly on body surface, which may minimize motion artifacts during body movements (Ren et al., 2018). On the other hand, for electrical stimulation, removing the interference of the stratum corneum also benefits the current conduction for precise stimulation (Motogi et al., 2016). Various fabrication methods for MAE have been proposed, including photolithography (Srivastava et al., 2015), laser machining (Omatsu et al., 2010), micro molding (Wang et al., 2022a), 3D printing (Krieger et al., 2019), etc., while they are either high-cost or inconvenient to perform. In recent years, a simple and low-cost approach named magnetization-induced self-assembly method (Pan et al., 2016) was proposed and successfully proved for MAE fabrication. The performances of MAE on electrophysiological recordings have been widely studied, where high-quality EMG and EEG signals can be acquired by using wearable MAE systems (Ren et al., 2016). However, most reported work focused mostly on materials and device fabrication, and the performance evaluation was usually not very comprehensive due to limited testing scenarios. In another aspect, the use of MAE for intuitive sensory feedback through electrical stimulation is seldom reported. As a result, a study on the performance of MAE for closed-loop HMI applications is necessary and valuable to provide technique support for future clinical applications of MAE.

In this study, we fabricated MAE samples with the easy-to-operate magnetization-induced self-assembly method. The fundamental characteristics of MAEs were compared with the commonly used metal-based flat dry electrodes with the same dimension. The performances of MAEs for the closed-loop HMI applications were thereafter evaluated, including the MAE-based EMG recording in the descending pathway with corresponding myoelectric motion-intention decoding, as well as the sensory feedback by the MAE-based transcutaneous electrical nerve stimulation (TENS) in the ascending pathway with corresponding somatosensory evoked potential (SSEP) analysis (i.e., EEG analysis). The results were discussed in detail and future improvement work was proposed.

## 2 Materials and methods

### 2.1 Sample fabrication

**FIGURE 1:**
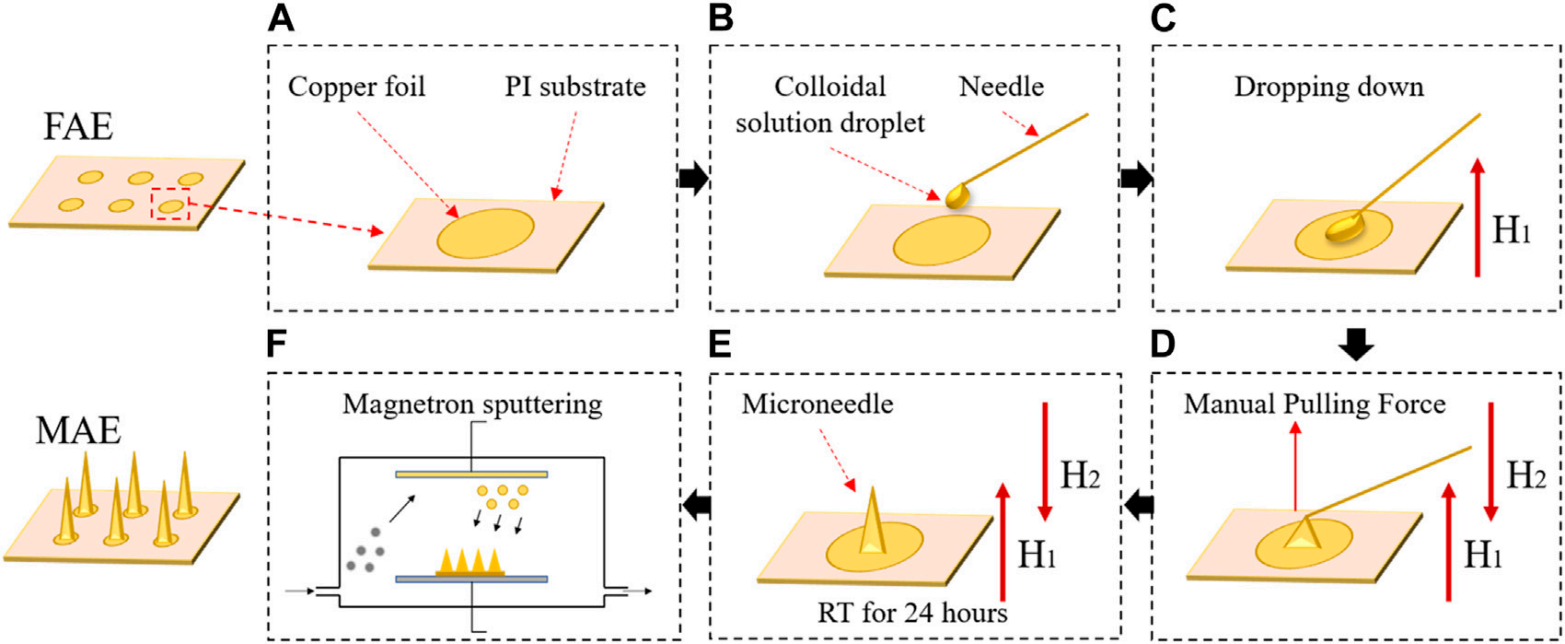
The fabrication procedure of a microneedle array electrode (MAE) sample: **(A)** a finished FAE; **(B)** the pre-prepared colloidal solution was manually dropped down; **(C)** a magnetic field was applied to produce a repulsive force on the magnetized colloidal solution droplets and each droplet was manually pulled to form microneedles by using a needle; **(D)** a second magnetic field was applied to produce an attractive force to the microneedles; **(E)** the repulsive and attractive force of the magnetic fields kept the newly formed microneedles suspending vertically until all the microneedles fully solidified; **(F)** a conductive composite film was deposited on the microneedle surface via the magnetron sputtering technology.

To ensure wearable applications of MAE, a flexible substrate is necessary. To this end, a non-toxic flexible polyimide film with a thickness of 300 μm was chosen as the substrate. Before the construction of microneedles, copper foils were firstly distributed on the substrate via the printed-circuit technique, and they all were electrically connected into one signal channel, forming a flat array electrode (FAE) (Figure 1A). The FAE was used as the inter-substrate for MAE fabrication and additionally used for performance comparison between MAE and FAE.

**FIGURE 2:**
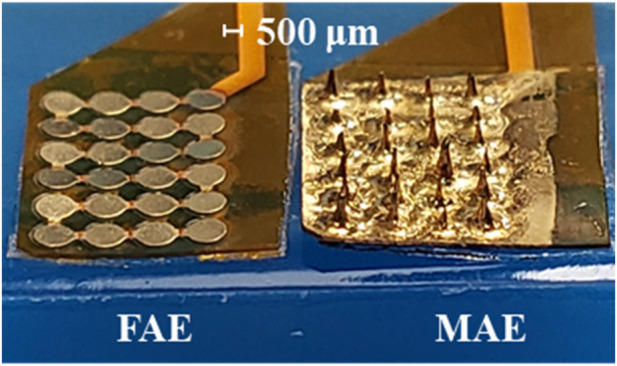
A finished flat array electrode (FAE) and microneedle array electrode (MAE) sample ready for testing.

Considering its easy manipulation and low cost in the lab, the magnetization-induced self-assembly method was selected to construct microneedles on the inter-substrate. The base material of microneedles was epoxy resin binder, which was a mixture of epoxy resin and curing agent by a volume ratio of 3:1. The epoxy resin binder was thereafter mixed with pure iron powders by a weight ratio of 1:0.7 and stirred evenly to achieve a magnetized colloidal solution. Then, the pre-prepared colloidal solution was manually dropped down onto each unit of the inter-substrate to form circular droplets with a diameter of ∼750 μm (Figure 1B). A magnetic field with an intensity of 0.2 T was applied perpendicular to the inter-substrate to produce a repulsive force on the magnetized colloidal solution droplets (Figure 1C). Then, each droplet was manually pulled to form microneedles by using a needle (Figure 1D). Afterwards, a second magnetic field of 0.2 T that was parallel to the first magnetic field but with opposite polarity was applied, producing an attractive force to the magnetized colloidal solution. The repulsive and attractive force of the magnetic fields kept the newly formed microneedles suspending vertically to the inter-substrate until all the microneedles fully solidified at room temperature in about 24 h (Figure 1E). Finally, a conductive composite film with 5 nm thick titanium and 100 nm thick aurum was deposited on the microneedle surface via the magnetron sputtering technology (Figure 1F). Figure 2 shows a finished MAE sample, and the detailed descriptions of the magnetization-induced self-assembly method have been introduced in the references (Ren et al., 2017; Chen et al., 2018; Ren et al., 2018).

The FAE samples prepared in this work assume a rectangular shape with 4 × 6 round electrode units, where each unit is with a diameter of 800 μm and a thickness of ∼35 μm, and the center-to-center distance between two adjacent units is 1,000 μm. The MAE samples have the same dimension as the FAE samples, and the microneedles are in cone shape and have a length of 500–600 μm and a tip diameter of ∼20 μm.

### 2.2 Subjects

Four subjects were recruited in the experiments, including three males and one female, with an average age of 32 ± 8 years, an average weight of 60 ± 10 kg, and an average height of 175 ± 5 cm. Preliminary medical examinations showed that all subjects were psychologically healthy to participate in this research. All subjects gave written informed consent and provided the permission for publication of photographs for scientific and educational purposes. The research protocol was approved by the Institutional Review Board of Shenzhen Institute of Advanced Technology, Chinese Academy of Sciences (*IRB Number: SIAT-IRB-190315-H0325*).

### 2.3 Measurement of impedance characteristics

In this work, the equivalent EII of MAE was simplified to an *RC* parallel circuit model (Villarreal et al., 2012), where the total impedance *Z* was calculated by using (1), the resistance *R* and capacitance *C* were measured with an impedance analyzer (*TH2829C, Tonghui Electronic, China*), and *f* is the frequency used in the fast-sweeping frequency method from 20 to 2000 Hz. To study the impedance characteristics, FAE and MAE samples were tested successively for performance comparison, where a pair of electrodes with a center-to-center distance of 4 cm was attached at the same position on each subject’s forearm. Five testing scenarios were designed to evaluate the impedance characteristics of electrode samples as follows, and every test was performed at least three times.

i) *Dry Condition:* Subjects sat calmly during the tests, and their skins were cleaned with 75% rubbing alcohol by using cotton swabs and dried before the test.
ii) *Wet Condition:* With cleaned and dried skins, subjects sat calmly during the tests, but additionally 0.2 mL drinking water was applied to the skin surface before the attachment of electrodes.
iii) *Pressure Condition:* With cleaned and dried skins, subjects sat calmly during the tests, but additionally with a weight of 500 g placed vertically above the electrodes.
iv) *Dynamic Condition:* With cleaned and dried skins, subjects continuously swung their tested arm during the tests.
v) *Motion Condition:* With cleaned and dried skins, subjects continuously closed and opened their hands to keep the tested muscle contracting during the tests.

$$Z=\frac{R}{\sqrt{1+{\left(2\pi fCR\right)}^{2}}}$$ (1)

### 2.4 EMG recording and motion-intention decoding

**FIGURE 3:**
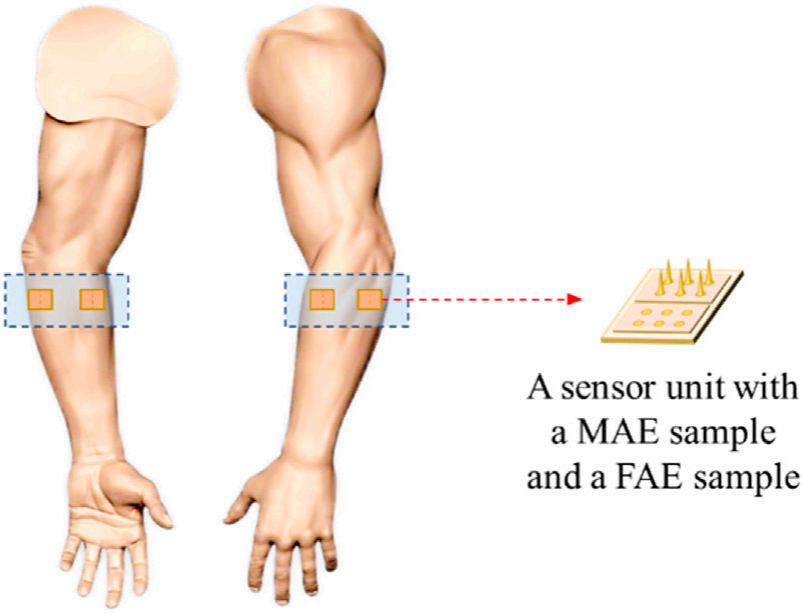
Electrode placement for EMG and EEG recording.

For EMG recording, a wristband with four-channel MAE and four-channel FAE was prepared. As shown in Figure 3, four MAEs distributed equally on the wristband, and additionally, a FAE was attached next to each MAE. The wristband was connected to a multichannel EMG acquisition system (*NES-16A01, SIAT, CAS, China*) and signals were collected with a sampling rate of 2000 Hz. During signal acquisition, subjects performed six classes of dynamic hand motions, including hand close (HC), hand open (HO), wrist extension (WE), wrist flexion (WF), forearm supination (FS), and forearm pronation (FP), as well as relax (RE), with three levels of strength of maximum (MAX), medium (MID), and minimum (MIN). Two scenarios were designated, i.e., the *Static Condition* where subjects sat calmly on a chair and performed the motions, and the *Dynamic Condition* where subjects swung their tested arms and performed the motions simultaneously. Each motion class was executed three times and a 5-s rest was adopted between neighboring motion executions to avoid muscle fatigue. An extra 30-s rest was taken before switching to the next motion class.

The EMG data were preprocessed and analyzed with *Matlab 2018b*. A bandpass filter with a range from 10 to 500 Hz and a notch filter with a frequency of 50 Hz was utilized to filter out motion artifacts and power frequency interference caused by the electronic circuit. The signal-to-noise ratio (SNR) of EMG signals acquired with MAE and FAE was calculated by using (2), where RMS means the root-mean-square value, Vsignal and Vnoise are the amplitude of signal and noise, respectively. For motion-intension decoding, a sliding window method (Li et al., 2017) was used with a window length of 150 ms and an increment of 100 ms, and time-domain features including absolute average value (MAV), wavelength (WL), zero-crossing rate (ZC), and root-mean-square (RMS) were extracted from the filtered EMG signals. The linear discriminant analysis (LDA) algorithm (Li et al., 2022) was used to train a classifier, which was thereafter used to calculate average classification accuracy (Phinyomark et al., 2013).

$$SNR=20\times \mathrm{lg}\frac{RMS\left(V_{signal}\right)}{RMS\left(V_{noise}\right)}$$ (2)

### 2.5 Electrical stimulation and EEG analysis

Several studies demonstrated that the TENS applied on the skin surface corresponding to some nerves would generate not only the feelings of stimulated positions but also some intuitive perceptions of other body parts (Geng et al., 2018). The former is called direct feeling and the latter is called indirect feeling, and different feeling types can be realized by adjusting stimulation parameters. Additionally, the indirect feelings can be evoked in upper-limb amputees, where electrical stimulation properly applied on their residual limbs can restore perceptions of the lost hands even including distinct fingers, i.e., the phantom finger perception (Chai et al., 2013). The perception evoking, in other words, the sensory feedback, is an indispensable part of many closed-loop HMI applications, and electrodes always play an important role since they strongly determine the quality of TENS.

**FIGURE 4:**
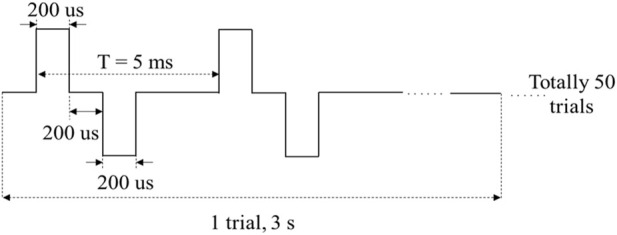
The pulse pattern for electrical stimulation.

To assess the performance of MAE on TENS for sensory feedback application, an electrical stimulation platform was set up, which consisted of a pair of MAEs to form a stimulation pathway, an isolated bipolar constant current stimulator (*DS5, Digitimer, United Kingdom*), a waveform generator (*CED Micro1401-4, Digitimer, United Kingdom*), and a computer for parameter adjustment. According to the results of our previous study on sensation evoking (Wang et al., 2022b), bipolar square waves were selected in this work and applied on different positions of subjects’ wrist area, i.e., the corresponding positions of radial nerve, median nerve, and ulnar nerve, and on subject’s middle finger pulp as well, through the MAE pair that had 1 cm in between. Table 1 summarizes the stimulation parameters varied in the experiments for sensation evoking. Figure 4 shows the pulse pattern for the stimulation, where each trial lasted for 3 s, including two pairs of bipolar square waves with a period of 5 ms, and the pulse width was 200 µs with an interval of 200 µs. Totally 50 trials were applied for each stimulation, and a 5-min rest was designated before switching to next stimulation. During the stimulations, subjects sat calmly on a chair and reported the position, strength, and type of sensations they felt. Subjects scored the sensation strength according to their subjective feelings in a range from 0 to 10, where 0 indicated no sensation and 10 indicated the sensation was extremely uncomfortable.

**TABLE 1:** The parameters for electrical stimulation.

Amplitude (mA)	0, 0.5, 0.75, 1, 1.25, 1.5, 1.75
Frequency (Hz)	5, 50, 100, 200
Wave width (μs)	200
Interval time (μs)	200

EEG analysis was performed to evaluate the MAE-based sensory feedback. For EEG recording, A standard EEG signal acquisition system (*64-channel QuikCap; Amplifier: SynAmps 2, Neuroscan, USA*) is adopted. In the experiments, subjects were required to sit in a specially designed laboratory that was shielded from external electromagnetic interferences, and keep still to avoid any motion artifacts. The EEG signals were recorded with a sampling rate of 1,000 Hz, and automatically marked once the stimulation started. The recorded EEG data were preprocessed by the EEGLab toolbox in *MATLAB 2018b* (Delorme and Makeig, 2004). A bandpass filter with a range from 1 to 45 Hz and a notch filter with a frequency of 50 Hz was utilized to filter out artifacts such as the baseline drift as well as the power frequency interference. The Independent Component Analysis (ICA) algorithm was adopted to further process the filtered data to remove disturbances such as EOG and stimulus signal artifacts (Chaumon et al., 2015). Thereafter, the data segment from 200 ms before the stimulus to 1,000 ms after the stimulus was extracted, and the stimulus onset was set as point zero. Additionally, for each channel, 50 EEG data segments achieved upon 50 stimulation trials were superimposed and averaged to weaken the interference of spontaneous EEG (Lopez-Calderon and Luck, 2014).

## 3 Results

### 3.1 Impedance characteristics

**FIGURE 5:**
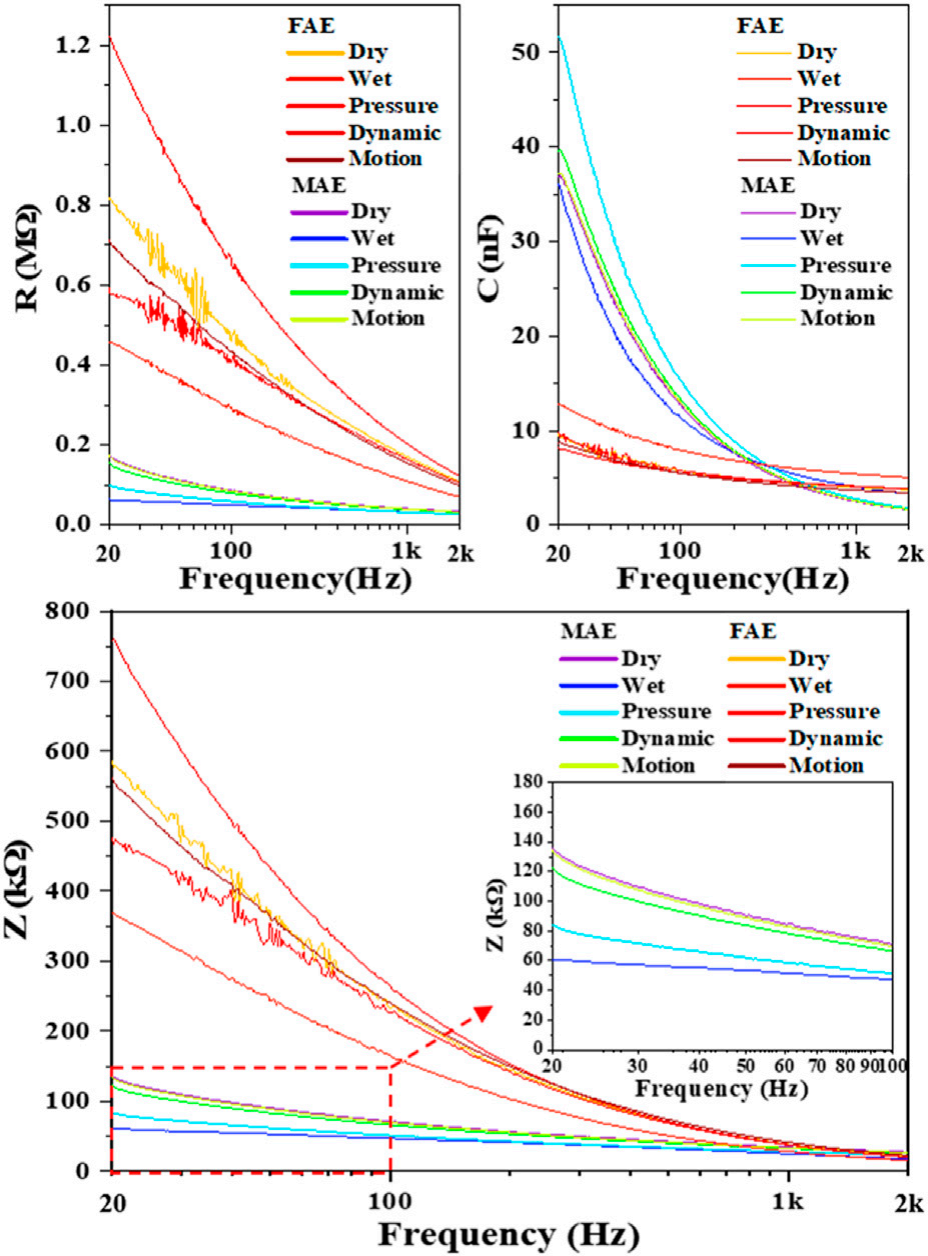
The *R*, *C*, and *Z* curves in the whole testing frequency range from 20 to 2000 Hz for a typical subject.

In assessment of electrode-skin interface impedance, the *RC* parallel circuit model is mostly used. The *R* and *C* represent the resistance and capacitance between electrodes and skin surface, respectively, and *Z* is the total impedance amplitude calculated with *R* and *C* values. Considering 20–2000 Hz is commonly used in sampling electrophysiological signals, we selected this range for testing in this work. Table 2 shows the average values and standard deviations of resistance *R*, capacitance *C*, and calculated impedance *Z* of MAE and FAE samples at the frequency of 20 and 2000 Hz across all the four subjects, which were acquired in the scenario of *Dry Condition*. As expected, at low frequency, the *R* and *Z* of MAE are nearly one order of magnitude smaller than those of FAE, and the *C* of MAE is larger than that of FAE; at high frequency, both MAE and FAE have very similar *R*, *C*, and *Z* values. Figure 5 shows the *R*, *C*, and *Z* curves in the whole testing frequency range from 20 to 2000 Hz for a typical subject, which were acquired in different scenarios of *Dry*, *Wet*, *Pressure*, *Dynamic*, and *Motion Conditions*, respectively. As can be seen, in all the testing conditions, the *R*, *C*, and *Z* amplitudes decrease largely with the increase of frequency for both MAE and FAE. The MAE illustrates significantly smaller *R* and *Z* but larger *C* than the FAE, especially at low frequencies. In addition, there exist obvious fluctuations on some of the EII curves for FAE at low frequencies from 20 to 100 Hz, which can be attributed to the slightly relative displacement between FAE and skin surface. Due to the well-fabricated microneedle structure of MAE, part of the needle length could penetrate into the epidermis, which reduces the probability of relative displacement between MAE and skin surface, and thus MAE always has very smooth curves in the whole frequency range.

**TABLE 2:** The average values and standard deviations of *R*, *C*, and *Z* of MAE and FAE samples across all the four subjects.

	FAE		MAE	
	20	2000	20	2000
R (kΩ)	2,560 ± 1,246	141 ± 24	298 ± 106	103 ± 60
C (nF)	5.8 ± 2.4	2.5 ± 0.9	23.3 ± 11.2	0.6 ± 0.7
Z (kΩ)	1,275 ± 462	34 ± 10	240 ± 93	94 ± 56

### 3.2 Performance on EMG recording and application

**FIGURE 6:**
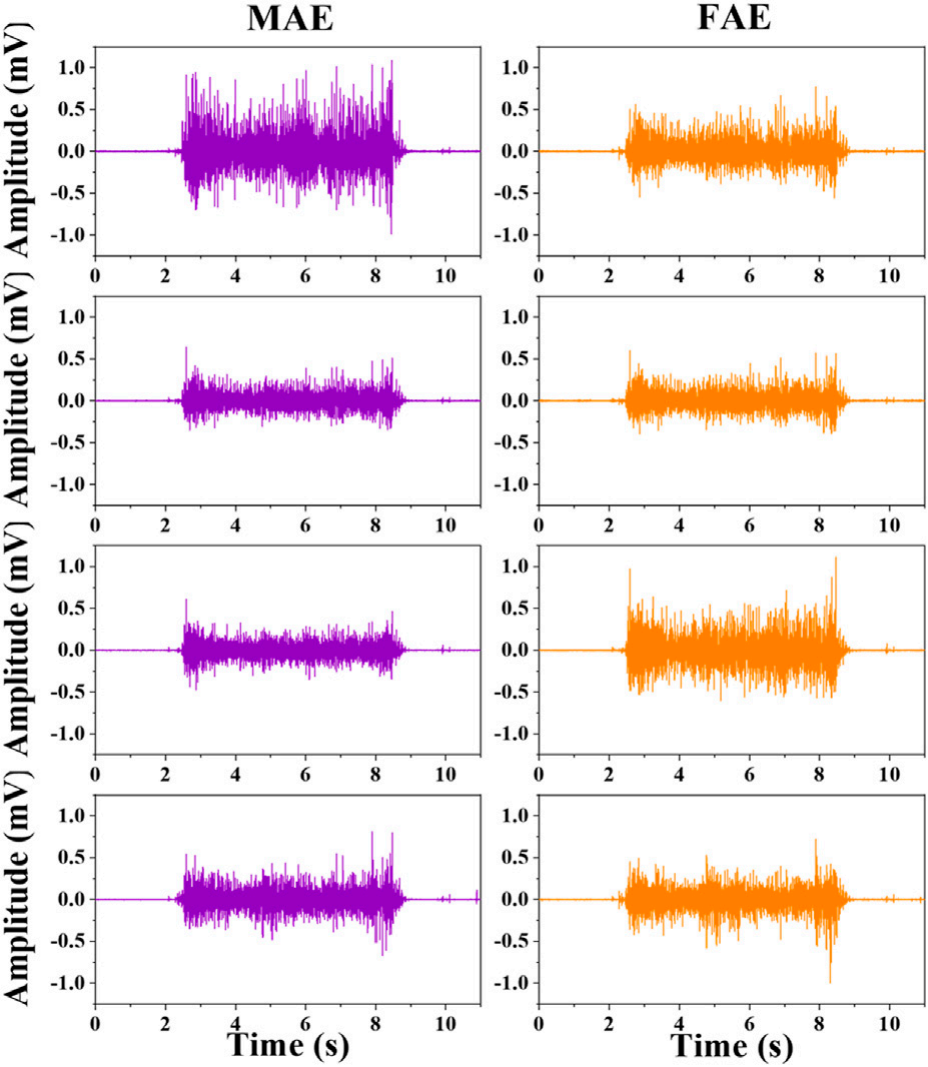
The typical four-channel raw EMG recordings acquired by MAE and FAE for hand-closing executed in the *Dynamic Condition*.

**FIGURE 7:**
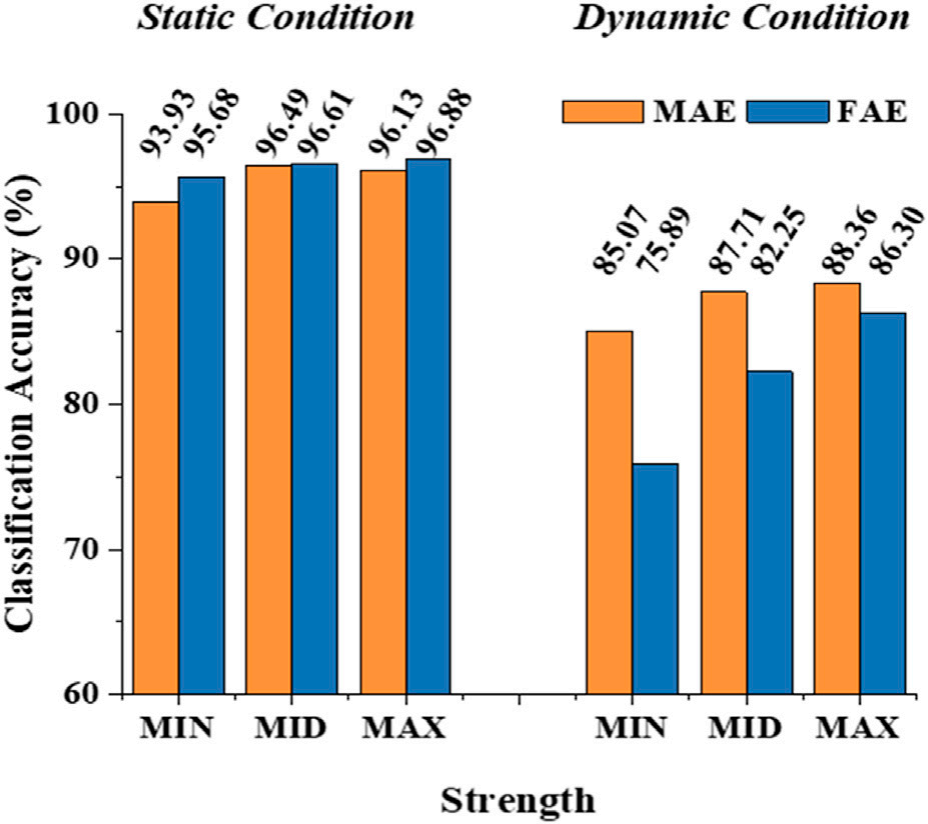
The classification accuracy of motion-intention decoding based on the EMG signals recorded with MAE and FAE samples.

Figure 6 shows the typic four-channel raw EMG recordings acquired by both MAE and FAE samples, for the movement of hand-closing executed in the scenario of *Dynamic Condition*. As can be seen, clear EMG signals can be recorded by both electrodes, but the MAE illustrates higher signal amplitudes than the FAE under the same condition, which is possibly due to the lower EII of MAE. Table 3 shows the averaged SNR of EMG recordings across all the hand movements and signal channels in both *Static* and *Dynamic Conditions*, where each movement was performed with different strength levels, respectively, and the results show that the SNR of MAE is always higher than that of FAE in all the testing conditions. Figure 7 shows the results of motion-intention decoding based on the EMG signals recorded with MAE and FAE samples, where the CAs were computed by using the LDA algorithm and averaged across all the movements. In the *Static condition*, there is no obvious difference between the CAs by MAE and FAE, no matter how strong the movement execution strength is. In the *Dynamic Condition* where subjects performed hand movements during arm swing, although the motion intention decoding is less accurate than that in the static scenario, the MAE can always achieve higher CAs than the FAE at all the strength levels. Moreover, the weaker the movement execution strength is, the superior the MAE compared to the FAE is.

**TABLE 3:** The averaged SNR of EMG recordings across all the hand movements and signal channels in the *Static* and *Dynamic Conditions*.

Condition	Strength	Sample	Average SNR (dB)	MAE vs FAE surpass rate (%)	
Static	MIN	MAE	23.7	39.4	Ave
		FAE	17.0		
	MID	MAE	31.8	32.5	
		FAE	24.0		
	MAX	MAE	44.2	21.4	
		FAE	36.4		
Dynamic	MIN	MAE	15.0	33.9	Ave. 36.0
		FAE	11.2		
	MID	MAE	20.6	42.1	
		FAE	14.5		
	MAX	MAE	24.8	31.9	
		FAE	18.8		

### 3.3 Performance on TENS for sensory feedback

**FIGURE 8:**
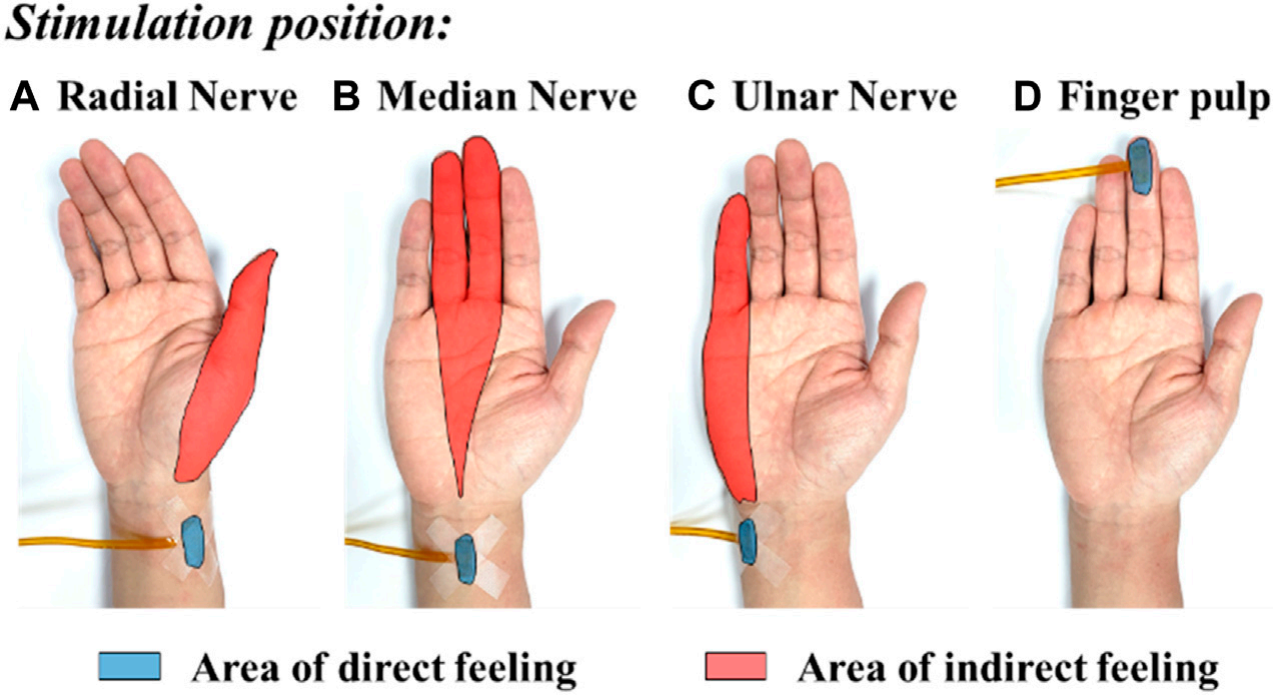
The typical mapping relation of sensation evoking by using the MAE-based TENS applied on different positions of the subject’s wrist and finger pulp: **(A)** radial nerve, **(B)** median nerve, **(C)** ulnar nerve, and **(D)** finger pulp.

**FIGURE 9:**
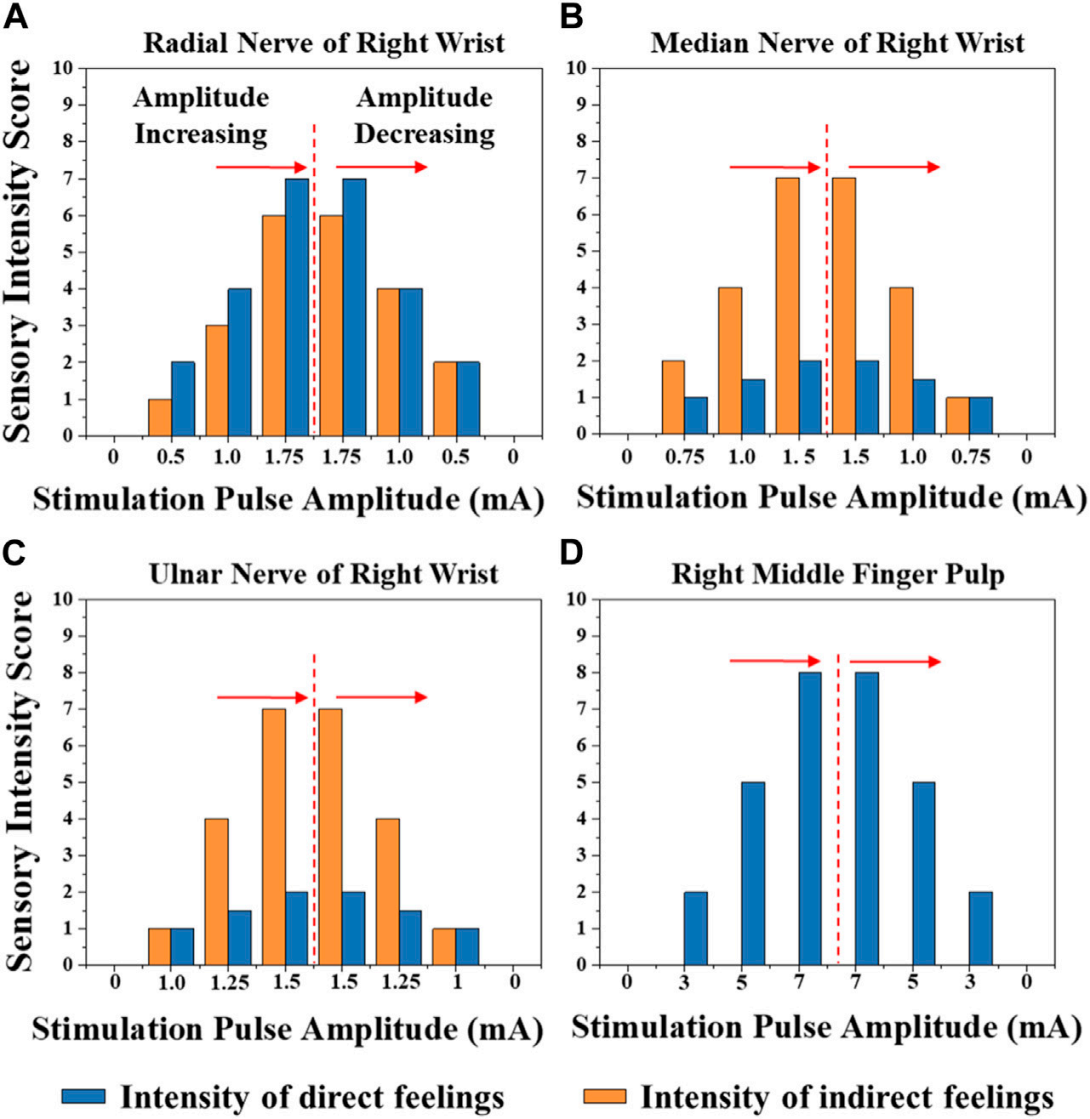
The intensities of the direct and indirect feelings correlated to the stimulation amplitude applied with MAE on **(A)** radial nerve of right wrist, **(B)** median nerve of right wrist, **(C)** ulnar nerve of right wrist, and **(D)** right middle finger pulp, when the amplitude increases and decreases.

Figure 8 shows the typical mapping relation of sensation evoking in a subject, by using the MAE-based TENS for sensory feedback in HMI application, where in (a), (b), and (c) the stimulations are applied on different positions of the subject’s wrist area, and direct feelings of stimulated points and indirect feelings of fingers and palm are reported by the subject; in (d) the stimulation is applied on the subject’s middle finger pulp and only direct feelings of the stimulated point are reported. As can be seen, for the illustrated subject, stimulation on the radial, median, and ulnar nerve not only generate direct feelings of the stimulated point, but also evoke intuitive feelings of the thumb finger, middle and ring fingers, and little finger in the subject’s brain, respectively. In addition, sensations of different parts on the palm that connect to the corresponding fingers, i.e., the thenar, the center of the palm, and the hypothenar, are evoked simultaneously. However, stimulation on finger pulp can only generate direct feelings of the stimulated point. The results in other subjects show similar sensation-evoking patterns as Figure 8, i.e., stimulation with MAE on a nerve can evoke indirect but intuitive feelings of the finger(s) and palm area which are innervated by the nerve. Figure 9 indicates that the intensities of the direct and indirect feelings are positively correlated to the stimulation amplitude applied with MAE when the amplitude increases and decreases. Table 4 shows that by adjusting the stimulation frequency, different sensation types can be induced for both direct and indirect feelings, where sensations of tapping, coherent tapping, and vibration are reported by the subject when the frequency is increased from 5 to 200 Hz.

**TABLE 4:** Sensations induced with different stimulation frequencies.

Stimulation parameters		Type of sensation
Frequency (Hz)	Wave width (us)	
5	200	Tapping
50		Coherent tapping
100		Vibration
200		Vibration

**FIGURE 10:**
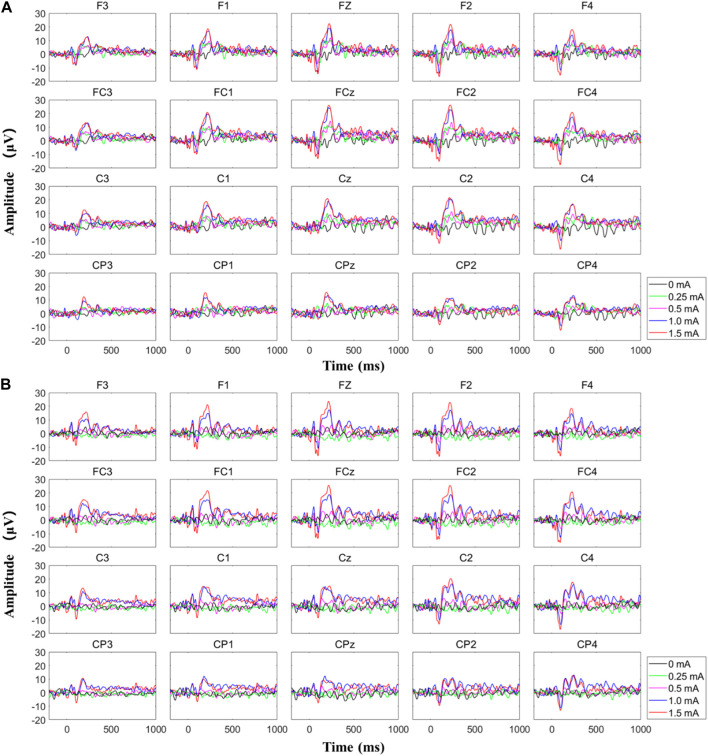
The typical EEG signals for a subject when MAE-based electrical stimulation is applied to the subject’s median nerve to induce indirect feelings for sensory feedback, where **(A)** the stimulation amplitude increased from 0 to 1.5 mA and **(B)** the stimulation amplitude decreased from 1.5 to 0 mA.

Figure 10 shows the typical EEG signals, i.e., the SSEP recordings, for a subject when MAE-based electrical stimulation is applied to the subject’s median nerve to induce indirect feelings for sensory feedback (refer to Figure 8B, with a stimulation frequency of 200 Hz). As illustrated in Fig. 10(a), with the increase of stimulation amplitude from 0 to 0.25 mA when there is no feeling of stimulation reported by the subject, no event-related potential (ERP) peak is generated on the EEG recordings. With a further increase of amplitude to 0.5 mA, a very tiny peak with a latency of around 236 ms can be observed. If the amplitude is increased to 1 and 1.5 mA when the subject can feel significant indirect feelings of vibration, obvious ERP peaks can be seen with a latency of around 215 and 220 ms. Fig. 10(b) indicates a confirmatory test with the same parameters in the same subject, but the stimulation amplitude decreases from 1.5 to 0 mA. Similarly, clear ERP peaks are generated when significant feelings are reported by the subject with stimulation amplitude of 1.5 and 1 mA, and the corresponding ERP latency is 222 and 225 ms, respectively. The average value and standard deviation of ERP latency for the MAE-induced sensory feedback in the illustrated subject is 220.5 ± 3.6 ms, and the result across all the four subjects is 224 ± 26.5 ms, which is in agreement with previous studies (Wang et al., 2022b). This results objectively demonstrate the effectiveness of MAE-based TENS for sensation evoking.

## 4 Discussion

High-quality electrophysiological signal acquisition is widely used in health monitoring, early disease prevention, HMI systems and so on. Usually, the quality of signals recorded with surface electrodes, including the wet electrodes and metal-based flat dry electrodes, can be affected by various factors such as EII, electrode displacement during body movements, long-term sustainability, etc. Although implantable acquisition techniques like needle electrodes may realize accurate signal recording, however, this invasive method can much more likely harm the human body and owns very low acceptance from the users. Thus, the MAEs, as a neural interface balancing the high signal quality and low injury to human body, have drawn more and more interests from both research and application disciplines. MAEs can build a direct and stable connection between the external electric circuit and the epidermis that contain useful electric signals, by avoiding the influence of the stratum corneum and simultaneously without touching the dermis that contains blood capillaries and nerve endings.

The refined microneedle structure is very critical to ensure the performance of MAEs, which also strongly depends on the sample fabrication method. Compared with other fabrication techniques such as laser cutting, 3D printing, micro molding, etc., the magnetization-induce self-assembly method benefits several strong points like low cost and simple operation procedure. The photos of MAE samples prepared in this work demonstrated the promising microstructure, where regular needles distribute equally and remain perpendicularly on the substrate. The signal acquisition results also prove the successful structure building with the method adopted in this work. During the experiments, the microstructure was stable and sustainable for several testing rounds, depending on different application scenarios. Although the low-cost MAEs are initially designed for single use, the reusable feature would further make them more competitive for clinical applications. During and after the experiments, there was no report or observation of bleeding, inflammation, pain, or any other uncomfortable feeling in all the subjects, and the indentation caused by pressure disappeared soon after the release of electrodes. This phenomenon indicates that the needle tips of MAEs do not touch the dermis. As reported in (van der Maaden et al., 2012; Nishinaka et al., 2013; Benitez and Montans, 2017), the stratum corneum of human skin is about 15–20 μm thick and the epidermis is about 130–220 μm thick. Considering the length of microneedles prepared in this work is about 550 μm, it can be concluded that only part of the microneedle length penetrated the subjects’ skin. Actually, from the photos, it can be seen that the microneedles have a cone shape, and the diameter becomes larger and larger from the needle tip to base part, which can prevent the penetration of the entire needle length into the skin and ensure the safety of MAEs.

The measurement of impedance characteristics shows that the EII of MAEs is consistently lower and more stable than that of FAEs in the whole testing frequency range, and the EII curves of MAEs are quite smooth under all the testing conditions. The results confirm the direct signal path between the external electrical circuit and the internal electrophysiological signal source constructed with MAEs, by avoiding the high impedance of stratum corneum and the influence of varying skin surface environments. In addition, the penetration of microneedles can fix the whole electrode tightly on the skin surface and prevent the displacement of the electrode, which would limit the motion artifact and improve the quality of the signal recording, especially in various dynamic scenarios.

With the stable microneedle structure and low EII, as expected, MAEs can achieve higher average SNR of EMG signals than FAEs, which is 31% higher in the *Static Condition* and 36% higher in the *Dynamic Condition*. The enhancement of SNR in the *Dynamic Condition*, in which subjects swung their tested arms and performed the motions simultaneously with different strength levels, may further highlight the signal recording performance of MAEs in actual applications. As a result, it is deserved that MAEs can realize more accurate motion-intention decoding than FAEs in the *Dynamic Condition*. When motions are performed during arm swing, the motion artifact caused by the electrode displacement is largely increased for FAEs, but MAEs can still retain stable signal recording and relatively accurate motion classification. It is also found that for the motions executed with weaker strengths, the difference in CA between MAEs and FAEs is more obvious and can be as high as 12% (85.07% over 75.89%). In the *Static Condition*, the stable attachment of FAEs is not influenced by motion execution and thus FAEs can also obtain accurate motion classification that is similar to MAEs.

In this work, MAEs are verified not only for the recording of descending control signals from human brain to machine, i.e., the EMG recording, but also for the stimulation of ascending feedback signals from machine to human brain, i.e., the TENS-based sensory feedback, to construct a bidirectional information transmission for closed-loop HMI application. The sensory feedback by electrical stimulation has been realized in several previous researches, with promising potentials in reestablishing sensory function for patients with sensory disturbance, or building up sensory feedback on some special occasions, but usually with traditional electrodes. Upon the dehydration of gels, the EII of wet electrodes changes and it is hard to ensure stable electrical stimulation parameters by using wet electrodes. On the other side, the relatively high EII of metal-based flat dry electrodes would generate a relatively high voltage across the electrode-skin interface upon a small current, which may cause an uncomfortable feeling like pricking. By combining the strong points of dry electrodes, e.g., stable EII and easy-to-use, and wet electrodes, e.g., low EII and limited relative displacement during movements, MAEs are suggested to enable effective, accurate, and safe electrical stimulation for sensory feedback. As proved by the experiments, not only the direct feelings of stimulated points on the wrist but also the indirect but intuitive feelings of the fingers and palms are successfully evoked in the subjects’ brains by using MAE-based electrical stimulation. The mapping relation between the position of stimulation (the areas of radial, median, and ulnar nerve) and the position of sensation evoking (the thumb, middle, ring, and little fingers, as well as the thenar, palm center, and hypothenar) is very clear. In addition, by changing the amplitude and frequency of stimulation current, it is possible to adjust the intensity and type of evoked sensations (touch, continuous tap, vibration, and pressure) across all the subjects. The successful sensation evoking can demonstrate the effective TENS by using MAEs, which is also benefited from the direct and stable connection between the external electric circuit and the subcutaneous layer constructed by the microneedles. What is more, by using MAEs, the current amplitude can be as small as 0.5 mA to evoke distinct sensations and an increase of 0.25 mA will cause an obvious change in sensation intensity. This phenomenon indicates the accuracy of the MAE-based TENS, compared with the stimulation through hydrogel electrodes which was studied in our previous work (Wang et al., 2022b), where a minimum amplitude of 3 mA is required to evoke a distinct sensation under the same experimental circumstance. The EEG recordings can objectively verify the effectiveness of the afferent pathway for sensory feedback established with the MAE-based TENS, where SSEP signals with clear ERP peaks are generated upon the subjective sensory feelings reported by the subjects.

Nevertheless, there are still some deficiencies that should be overcome for future clinical applications. During the experiments, although the MAE samples were reusable, their lifetime is still unsatisfactory, with a deterioration of signal quality after serval rounds of tests. The most problem is the fracture of microneedles that is caused by improper insertion of needles into the skin, where a transverse force perpendicular to needles can break off the needles easily. Thus, an enhancement of the structure strength of microneedles is suggested, possibly by changing the material type and/or ratio for microneedle fabrication. Another limitation is the exfoliation of conductive layer on the microneedle surface that is deposited via the magnetron sputtering technology. A tight bonding of conductive layer on microneedle surface should be considered, for example, with an extra adhesion layer or via some other coating or manufacturing techniques. Although this work has preliminarily demonstrated the promising performance of MAEs as an effective neural interface, for possible HMI applications including high-quality electrophysiological recording and accurate electrical stimulation for sensory feedback, there still exist some aspects relating to practical applications that should be explored in detail, as being studied and will be reported in our future publications.

## 5 Conclusion

This work fabricated a kind of microneedle array electrode (MAE) by using the magnetization-induced self-assembly method. The regular microneedles can penetrate through the stratum corneum and reach the epidermis, but not touch the dermis, establishing a safe and direct connection between the external electrical circle and internal electrophysiological signal source. MAEs show significantly lower electrode-skin interface impedance and higher signal-to-noise ratio compared with the normal metal-based flat dry electrodes, and achieve better performance on motion-intention decoding, especially in dynamic scenarios. In addition, the high-accuracy transcutaneous electrical nerve stimulation by using MAEs can evoke distinct and intuitive sensory feedback in subjects, which is objectively verified by EEG analysis. This work demonstrated the performance of MAEs working as a neural interface, on both electrophysiological recording and electrical stimulation, which may enable lots of potential human-machine interaction applications.

## Data Availability

The original contributions presented in the study are included in the article/supplementary material, further inquiries can be directed to the corresponding authors.
